# A nested case-control study indicating heavy metal residues in meconium associate with maternal gestational diabetes mellitus risk

**DOI:** 10.1186/s12940-015-0004-0

**Published:** 2015-02-28

**Authors:** Siyuan Peng, Liangpo Liu, Xueqin Zhang, Joachim Heinrich, Jie Zhang, Karl-Werner Schramm, Qingyu Huang, Meiping Tian, Syed Ali Musstjab Akber Shah Eqani, Heqing Shen

**Affiliations:** Key Laboratory of Urban Environment and Health, Institute of Urban Environment, Chinese Academy of Sciences, Xiamen, 361021 PR China; Xiamen Maternity and Child Health Care Hospital, Xiamen, 361003 PR China; Institutfür Epidemiologie I, Helmholtz Zentrum München - German Research Center for Environmental Health (GmbH), Ingolstädter Landstr. 1, 85764 Neuherberg, Germany; Molecular EXposomics (MEX), Helmholtz Zentrum München - German Research Center for Environmental Health (GmbH), Ingolstädter Landstr.1, 85764 Neuherberg, Germany; Department für Biowissenschaftliche Grundlagen, Technische Universität München, Weihenstephaner Steig 23, 85350 Freising, Germany; Public Health and Environment Division, Department of Biosciences, COMSATS Institute of Information and Technology, Islamabad, Pakistan

**Keywords:** Heavy metals, Meconium, Gestational diabetes mellitus, China

## Abstract

**Background:**

Environmental pollutant exposure may play certain roles in the pathogenesis and progression of diabetes mellitus including gestational diabetes mellitus (GDM). We hypothesize that heavy metal exposure may trigger GDM during pregnancy. The objective of this study was to investigate the possible associations between selected heavy metal exposure and GDM risk.

**Methods:**

This investigation is a retrospective case–control study nested within a cohort of 1359 pregnant women. These participants were recruited in Xiamen Maternity and Child Care Hospital, China, during June to July, 2012. All their newborns’ meconium samples were collected. By reviewing the antenatal care records, 166 GDM mothers were screened out from the 1359 participants; 137 of 166 GDM mothers offered their newborns’ meconium samples for the metal analysis. Those 137 mothers were set as the case group. Similarly, 294 healthy mothers without any gestational complication were initially screened out from the rest 1193 non-GDM mothers. 190 of the 294 healthy mothers offered their newborns’ meconium samples for the metal analysis. Those 190 mothers were set as the control group. Arsenic (As), mercury (Hg), lead (Pb), cadmium (Cd), and chromium (Cr) levels in these case–control meconium samples were measured by inductively coupled plasma mass spectrometry. The possible association between the metal levels and maternal GDM risk of studied subjects was assessed by binary logistic regression.

**Results:**

GDM prevalence of 12.21% was observed in the investigated 1359 participants. The concentrations of As, Hg, Cr and Cd in studied cases were significantly higher (*p < 0.05*) than those of controls. After adjustments for maternal age, pre-pregnant body mass index, gravidity, parity, hepatitis B virus infection, and newborn sex, As, Cd and Cr were found to be positively associated with GDM prevalence in dose-dependent manners. Among them, As was detected in all samples and its levels associated the maternal GDM with the adjusted odds ratios of 3.28 [95% CI 1.24, 8.71], 3.35 [95% CI 1.28, 8.75] and 5.25 [95% CI 1.99, 13.86] for the 2^nd^, 3^rd^ and 4^th^ quartiles, respectively.

**Conclusions:**

The present work implies that exposure to some of the selected metals (noticeably As) may contribute to maternal GDM risk during pregnancy.

**Electronic supplementary material:**

The online version of this article (doi:10.1186/s12940-015-0004-0) contains supplementary material, which is available to authorized users.

## Background

It has been reported that a dramatic rise of worldwide diabetic people is expected to occur up to 366 million in forthcoming 30 years and preventative actions are needed to take to tone down this global issue [1]. In China, approximately 40 million people are suffering from type 2 diabetes (T2DM) [2]. Gestational diabetes mellitus (GDM) is defined as the symptom of the impaired glucose tolerance (IGT) with the onset during pregnancy [3] and some reports also documented its high prevalence rate in China [2]. GDM is an endocrine and metabolic disease and occurs when a woman’s pancreatic function is not sufficient to overcome the diabetogenic environment of pregnancy [4], which has been considered as a ‘pre-diabetic’ state and its pathophysiology is clearly related to T2DM [5]. It has become one of the predictors of T2DM in the later life of the mothers and their offspring by playing a key role in the rapidly increasing diabetes incidence via the unknown reason of ‘diabetes begetting diabetes’ [2]. Several definite traditional risk factors associated with the development of GDM includes maternal age, obesity, history of macrosomia, and strong family history of diabetes [3,4].

Besides the changes in human lifestyle and behavior (e.g. poor diet, lack of exercise) and the genetic predisposition [6], recently environmental pollutants have been suggested to pose additional risk to diabetes development [7-9]. Heavy metals have been described to exhibit some diabetogenic properties [7,8]. Exposure to mercury (Hg), lead (Pb), cadmium (Cd) and the metalloid arsenic (As) have been linked to the increasing incidence of T2DM by several reports [10,11]. The fact can be further explained by considering the example from Arseniosis-epidemic areas of Taiwan and Bangladesh [12], where the authors clearly observed the striking association between arsenic burdens and incidence of diabetes mellitus. Furthermore, exposure to these heavy metals during pregnancy may impact the sensitive hormonal activities and ultimately increase the risk of GDM by favoring the shift towards diabetes (i.e., diabetogenic environment of pregnancy) [2]. Hence, the scenario encouraged metabolic disruption during pregnancy [13], and supports the hypothesis that maternal exposure to heavy metals during pregnancy may contribute to higher GDM risk.

The need to check the prenatal exposure to pollutants has led the scientists to various bio-monitoring strategies involving the use of newborn meconium. Meconium, being the first stools of the newborn, offers several advantages to assess the prenatal exposure of wide range environmental contaminants [14]. The potential of using meconium is primarily due to its formation accompanied with the fetus growing as early as the 12^th^ week of gestation and its accumulation till the child birth, so that long-term exposure (i.e., over the last two trimesters of pregnancy) of different environmental pollutants including heavy metals can be signaled by associated maternal response [14-16]. In contrast to some traditional matrices such as urine, cord blood and amniotic fluid, which have several limitation e.g., ethical issues, difficult to collect and reflect only transient and/or recent exposure of pollutants, meconium also offers advantages including ease in sampling and non-invasive analysis [17]. In addition, it acts as a promising mutual sample to indicate both the prenatal and maternal exposure, and it also contained relatively more pollutant burden than other tissues, hence easier to detect and quantify. On the other hand, some studies had also addressed association between pollutant exposure and neonatal health outcomes by meconium analysis [14,17], but few studies addressed the maternal health condition like GDM.

However in the last decade, strong disagreements have arisen between supporters and opponents of the role of environmental chemicals in the developing diabetes observed in several regions of the world [7,8,12]. The link between GDM prevalence and environmental chemical exposure is also hampered by the complexity of the chemical mixtures present into the environment, both in terms of the numbers of compounds and the mechanisms by which they can affect subjects. Furthermore, most of epidemiological studies have been conducted from high contaminated area in order to study the effects of toxic metals [12], whereas trace metal toxicity due to different exposure routes and environmental conditions are ambiguous and need to be confirmed. Hence keeping in view the mentioned hypothesis of GDM risk and its linkages with heavy metals burdens, we designed a retrospective case–control study nested within a cohort of 1359 participants to investigate the associations between gestational exposure to some heavy metals and GDM risk. Paired mother-newborn clinical records of recruited subjects were collected and heavy metals of prime environmental concern and potential diabetes risk [10,18], i.e. chromium (Cr), Cd, Hg, Pb and metalloid As were assessed in their meconium samples based on case–control approach, to study the possible association of heavy metals and GDM risk.

## Methods

### Participant recruitment and GDM screening

**Figure 1:**
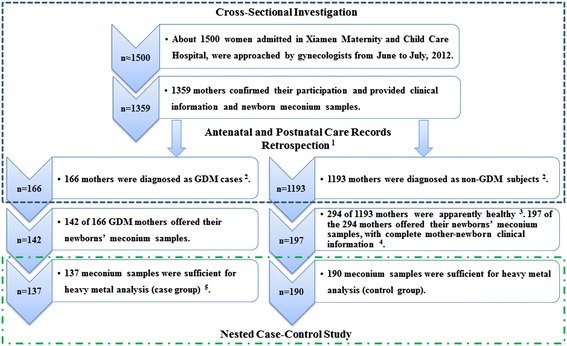
**Flowchart of the participant recruitment and case–control selection.** ^1^Clinical information of the participants was acquired from antenatal and postnatal care record retrospection, including all maternal gestational complications and neonatal disorders (Additional file 1: Table S2). ^2^GDM and non-GDM mothers were diagnosed during the 24 to 28^th^ gestational week. GDM cases were selected out through antenatal care record retrospection. ^3^Apparently healthy mothers without any recorded gestational complication were selected out from the 1193 non-GDM mothers (i.e., any complication potentially related to GDM was excluded), so the final controls had no GDM risk. ^4^Complete mother-newborn clinical information includes: A mother’s basic clinical characteristics (i.e. age, pre-pregnant BMI, gestational age, gravidity, parity, mode of delivery), gestational complications, history of drinking and smoking during pregnancy, history of macrosomic infant delivery, history of diabetes or obesity of herself and her spouse, and her newborn’s sex, birth weight and neonatal disorders. ^5^Most clinical information of those 137 mothers was available, except that 36 of 137 cases missed the maternal age or pre-pregnant BMI information. Metals exposure data of all 137 samples were included in the final statistical analysis.

The basic work flow of participant recruitment and case–control selection has shown as Figure 1. The participants of approximate 1500 pregnant women were approached, when they were admitted in hospital and expected to delivery. They were enrolled by gynecologists 1–3 days before their delivery in Xiamen Maternity and Child Care Hospital (Xiamen, China), from June to July, 2012. The subjects were informed about the objective of the study and solicited to participate after providing written consents by the gynecologists. The right to refuse participation and the fact that this refusal would not have any consequence on the relation with their physician, as well as the obligation of providing clinical information and authentic self-reported historical information during pregnancy, was explicitly mentioned. A total of 1359 mothers (mean age 26.90 years, mean pre-pregnant body mass index (BMI) 21.05 kg/m^2^) confirmed their participation by signing the consent forms and they all received complete antenatal and postnatal care in hospital. Clinical information of the mother-newborns was acquired from antenatal and postnatal care records. In addition, history of smoking, drinking during pregnancy, previous disease history of all the participants and their spouses were interviewed and recorded by the gynecologists during antenatal care.

Among the 1359 confirmed participants, GDM cases were selected out through retrospection of the maternal antenatal care records. GDM screening is a universal medical program in China which is nationally required and performed for every mother who receives antenatal care in hospital, by performing the glucose tests during the 24 to 28^th^ gestational week. All participants accepted the test and GDM cases among them were diagnosed and recorded. Briefly, the GDM screening tests consisted of a 50 g 1-hour glucose test (glucose challenge test) and a further oral glucose tolerance test (OGTT) based on the World Health Organization (WHO) criteria [19]. Venous blood was taken 60 min after the ingestion of 200 mL of 25% glucose solution. Mothers, with a record of ≥ 7.8 mmol/L glucose at the initial screening were invited to undergo a 75 g 2-hour OGTT. Diabetes is defined as fasting serum glucose ≥ 7.0 mmol/L and/or 2-hour serum glucose ≥ 11.1 mmol/L, and impaired glucose tolerance (IGT) is defined as 2-hour serum glucose ≥ 7.8 and < 11.1 mmol/L and fasting serum glucose < 7.0 mmol/L. Women who had confirmed either diabetes and/or IGT are regarded as the GDM cases. One diagnosed pre-gestational diabetes case (self-report) was excluded. A total of 166 subjects were diagnosed and recorded as GDM cases in the recruited cohort of 1359 participants, with GDM prevalence of 12.21%.

### Sample collection and case–control selection

Newborn meconium samples from the recruited cohort were collected by using the diapers during the first two postnatal days. The diapers were sealed in the acid-washed containers and stored at −20°C till the further treatment.

The selection of the cases was as follows: (1) During the 24 to 28^th^ gestational week, mothers were diagnosed as GDM cases (166 subjects); (2) Newborn meconium samples were successfully collected (142 subjects); (3) Samples were sufficient for heavy metal residues analysis (137 subjects, set as the case group). The selection of the controls was as follows: (1) During the 24 to 28^th^ gestational week, mothers were diagnosed as non-GDM cases (1193 subjects); (2) Mothers were apparently healthy, without any recorded gestational complication (Additional file 1: Table S2) (294 subjects); (3) Complete mother-newborn information was available and newborn meconium samples were successfully collected (197 subjects); (4) Samples were sufficient for heavy metal residues analysis (190 subjects, set as the control group). For both groups, mothers had (1) no smoking and drinking history during pregnancy; (2) no previous delivery of the macrosomic infant (birth weight > 4 kg); (3) no self-reported diabetes or obesity history of themselves and their spouses. The basic workflow of this study is shown in Figure 1.

### Sample preparation and heavy metal analysis

Meconium samples were dried by a freeze dryer (Boyikang Corporation, Beijing, China) and ground into powder. Dry meconium (100 mg) was digested with a mixture of 1 mL 65% (v/v) HNO_3_ and 1 mL 30% (v/v) H_2_O_2_ in a water bath at 100°C for at least 5 h, and adjusted to a final volume of 5 mL with ultra-pure water. Spiked samples were also prepared in the same procedure to measure recoveries. More details about the sample preparation were described in Additional file 1: Text S1.

Heavy metal residues in meconium were determined by using Agilent 7500cx inductively coupled plasma mass spectrometry (ICP-MS) (Agilent Technologies, Santa Clara, CA, USA). The calibration curves, linear ranges (LR), relative coefficients (r^2^) of calibration curves, the limit of detections (LOD), and the recoveries were listed in Additional file 1: Table S1. The strict quality control and quality assurance (QC/QA) in the analytical procedure were also followed and described in Additional file 1: Text S2.

### Statistical analysis

#### Data imputation

Undetected values of metals were imputed using the default value of 1/2 limit of detection (LOD) when the detection frequency ≥ 90%; otherwise, missing values were excluded to avoid any statistical bias [20,21]. Therefore, missing values of Pb were imputed by 1/2 LOD while missing values of Cr and Cd were excluded from statistical analysis.

### Difference comparison

Independent sample *t*-test and non-parametric Mann–Whitney *U* test were applied to compare the differences between two groups, for normally distributed and non-normally distributed continuous variables, respectively. Fisher’s exact test was applied to compare difference for categorical variables with total observed frequency ≤ 40, otherwise, Pearson Chi-square test was applied.

### Dose–response relationship

The associations between heavy metal levels and GDM prevalence were examined using binary logistic regressions. The heavy metal levels were transformed into categorical variables based on quartile cutoffs of the controls. Values of Cr and Cd were also transformed into three categorical variables based on cutoff-points at 50% and 75% values of the controls. Trend analysis was evaluated by linear by linear association Chi-square [22,23]. Adjustments were made for six suspected prenatal GDM risk factors (i.e., maternal age, pre-pregnant BMI, gravidity, parity, hepatitis B virus (HBV) infection, and newborn sex). Adjustments were made for maternal age, pre-pregnant BMI, gravidity and parity because those factors have been associated with GDM [3-5]. Adjustments were also made for newborn sex, because hormonal activities during pregnancy may be affected by fetal sexual differentiation [24], which may also contribute to GDM risk. All the recorded gestational complications were listed and compared in this recruited cohort (Additional file 1: Table S2). HBV infection, umbilical cord disorders, and precipitate labor were found to be significantly different between GDM and non-GDM group. However, only HBV infection is a prenatal factor which has been associated with GDM [25,26] while umbilical cord disorders and precipitate labor are likely to be the GDM induced pregnancy outcomes. Therefore, adjustment was made for HBV infection.

The statistical analysis was performed with SPSS version 18.0 (SPSS Inc., Chicago, IL). P value of less than 0.05 was considered to be significantly statistical difference.

## Results

### Clinical characteristics of 1359 participants

The total 1359 confirmed participants included 166 GDM and 1193 non-GDM mothers. The general clinical characteristics were listed and compared in Additional file 1: Table S3. Among them, maternal age and pre-pregnant BMI were significantly different *(p < 0.05)* between GDM and non-GDM mothers, with lower gestational age and higher incidence of C-section (Caesarean section) (27.71%) in GDM group. The scenario suggested potential association between higher maternal age, BMI and GDM risk, which is in agreement with previous investigations [3-5]. Furthermore, the occurrence of maternal HBV infection (i.e., HBsAg positive, measured during gestation from 25 to 28^th^ week in antenatal care for each mother), and some pregnancy outcomes (i.e. umbilical cord disorders and precipitate labor) were significantly different between the GDM and non-GDM mothers (Details were shown in Additional file 1: Table S2), suggesting that HBV infection may be a GDM risk factor in this cohort, and GDM may has adverse effects on pregnancy outcomes.

### Clinical characteristics of subjects in nested case–control group

137 GDM cases from the 166 GDM mothers and 190 controls from the 1193 non-GDM mothers were selected for the heavy metal exposure analysis and GDM risk assessment. The maternal and newborn clinical characteristics were summarized in Table 1. All factors except newborn birth weight and sex showed significant difference between the cases and controls (Table 1). Given that fetal sexual differentiation and development may affect maternal hormonal activities during pregnancy [24], the maternal and newborn clinical characteristics between the selected cases and controls were also compared separately on newborn sex (see Additional file 1: Table S4). For the mothers who delivered boys, all maternal factors were significantly different between the case and control groups. While for mothers who delivered girls, the maternal age, gravidity, parity did not showed difference between the case and control groups. Therefore, fetal sex might play a certain role in maternal status during gestation.

**Table 1:** **Clinical characteristics of the mothers and newborns in nested case–control group** ^**a**^

	**Case (n = 137)**	**Control (n = 190)**	**p** ^**b**^
Newborn birth weight (kg)	3.29 ± 0.38	3.23 ± 0.30	0.080
Maternal age (year)^c^	27.85 ± 3.87	26.34 ± 2.64	<0.001^**^
Maternal pre-pregnant BMI (kg/m^2^)^d^	21.89 ± 2.92	20.59 ± 2.27	<0.001^**^
Gestational age (week)	39.18 ± 0.80	39.55 ± 0.92	<0.001^**^
Newborn sex			
Male	72 (52.55%)	93 (48.95%)	0.520
Female	65 (47.45%)	97 (51.05%)	
Maternal gravidity			
1	82 (59.85%)	134 (70.53%)	0.034^*^
2	29 (21.17%)	38 (20.00%)	
≥3	26 (18.98%)	18 (9.47%)	
Maternal parity			
1	114 (83.21%)	174 (91.58%)	0.049^*^
2	22 (16.06%)	16 (8.42%)	
≥3	1 (0.73%)	0 (0.00%)	
Mode of delivery			
Eutocia	92 (67.15%)	176 (92.63%)	<0.001^**^
C-section	39 (28.47%)	13 (6.84%)	
Aid delivery	6 (4.38%)	1 (0.53%)	
Maternal HBsAg			
Positive	24 (17.65%)	0 (0.00%)	<0.001^**^
Negative	112 (82.35%)	190 (100.00%)	

^a^Values of newborn birth weight, maternal age, pre-pregnant BMI and gestational age are expressed as mean ± SD.

^b^p values of maternal age, pre-pregnant BMI and gestational age were obtained by non-parametric Mann–Whitney *U* test, p values of newborn birth weight was obtained by independent sample *t*-test, while p values of maternal gravidity and parity, mode of delivery were obtained by Pearson Chi-square test, p values of HBsAg was obtained by Fisher’s exact test. ^*^ indicates p < 0.05, ^**^ indicates p < 0.01.

^c^Values from 101 subjects in case group were available.

^d^Values from 103 subjects in case group were available.

### Meconium heavy metal profiling in nested case–control group

The detection frequencies of As and Hg were all 100% in both groups. For Pb, Cd and Cr, the detection frequencies were 89.78%, 100% and 55.47% in case group, respectively; and 97.37%, 45.79% and 70% in control group, respectively. The levels of As (median = 49.75 and 37.78 ng/g in cases and controls, respectively), Hg (median = 34.40 and 28.66 ng/g in cases and controls, respectively), Cr (median = 56.43 and 25.96 ng/g in cases and controls, respectively) and Cd (median = 9.41 and 4.10 ng/g in cases and controls, respectively) in the meconium samples of the cases were significantly higher than those in the controls (*p < 0.05*). In contrast, Pb levels (median = 146.90 and 135.68 ng/g in cases and controls, respectively) were found at very high levels in the study subjects but without significant difference (*p > 0.05*). More details were described in Table 2. Therefore, As, Hg, Cr and Cd were suspected to induce maternal GDM risk.

**Table 2:** **Profiling of heavy metals in meconium (ng/g dry weight) of nested case–control group** ^**a**^

**Metal**		**Case (n = 137)**	**Control (n = 190)**	**p** ^**b**^
As	Detected Number (ratio)	137 (100.00%)	190 (100.00%)	<0.001^**^
	Median	49.75	37.78	
	IQR	32.51-82.42	23.98-64.56	
	Mean	60.81	45.15	
	SD	37.19	28.71	
Hg	Detected Number (ratio)	137 (100.00%)	190 (100.00%)	0.001^**^
	Median	34.40	28.66	
	IQR	25.62-47.36	20.42-40.30	
	Mean	41.27	37.56	
	SD	27.09	54.34	
Pb	Detected Number (ratio)	123 (89.78%)	185 (97.37%)	0.534
	Median	146.90	135.68	
	IQR	49.96-389.10	75.61-216.54	
	Mean	283.96	195.55	
	SD	393.04	216.32	
Cr	Detected Number (ratio)	76 (55.47%)	133 (70.00%)	<0.001^**^
	Median	56.43	25.96	
	IQR	18.19-116.45	10.13-53.33	
	Mean	91.75	47.99	
	SD	105.69	66.54	
Cd	Detected Number (ratio)	137 (100.00%)	87 (45.79%)	<0.001^**^
	Median	9.41	4.10	
	IQR	5.59-15.23	1.47-11.32	
	Mean	18.55	10.00	
	SD	35.44	17.48	

^a^Missing values of Pb were imputed by 1/2 LOD while missing values of Cr and Cd were excluded from statistics.

IQR: Inter-quartile range, 25%-75%.

^b^p value was obtained by non-parametric Mann–Whitney *U* test.

^**^ indicates p < 0.01.

### GDM risk assessment of the selected metals in nested case–control group

To address the potential relationships between maternal exposure to the selected metals and GDM risk, adjustments were made for six factors (i.e. maternal age, pre-pregnant BMI, gravidity, parity, HBV infection, and newborn sex). The adjusted results were shown in Table 3 and Additional file 1: Table S5, and the unadjusted results were presented in Additional file 1: Tables S6 and S7. Positive dose-dependent trends were observed for As, Cd and Cr. In comparison of the 1^st^ quartile, As levels associated the maternal GDM with the adjusted odds ratios (AORs) 3.28 [95% CI 1.24, 8.71], 3.35 [95% CI 1.28, 8.75] and 5.25 [95% CI 1.99, 13.86] for the 2^nd^, 3^rd^ and 4^th^ quartiles, respectively; Cd levels associated the GDM with AORs 16.87 [95% CI 4.19, 67.86] and 11.95 [95% CI 2.97, 48.04] for the 3^rd^ and 4^th^ quartiles, respectively. Cr level was significantly associated with GDM risk only for the 4^th^ quartile samples with AOR 4.48 [95% CI, 1.40, 14.31]. The observed AORs with high CI ranges for Cd and Cr may be attributed to many of the missed data for the control subjects at the low level. When Cd and Cr values were transformed to three levels with values below median as reference cutoff (Additional file 1: Table S5), Cd levels were associated the GDM with AORs 8.26 [95% CI 3.39, 20.12] and 5.82 [95% CI 2.39, 14.16] for the 2^nd^ and 3^rd^ cutoff, respectively. Cr level was significantly associated with GDM risk at only 3^rd^ cutoff with AORs 3.27 [95% CI 1.35, 7.93]. The significance of association for Cd and Cr kept at high levels. The trend for As, Cd and Cr was all significant under all circumstances.

**Table 3:** **Associations of heavy metals with adjusted odds ratio (AOR) of GDM in nested case–control group** ^**a**^

**Metal**	**Control and case**	**1st quartile (Reference)**	**2nd quartile**	**3rd quartile**	**4th quartile**	**p trend** ^**b**^
As	Control (%)	47 (24.74%)	48 (25.26%)	48 (25.26%)	47 (24.74%)	
	Case (%)	11 (8.03%)	32 (23.36%)	44 (32.12%)	50 (36.50%)	
	AOR (95% CI)^c^	1	3.28 (1.24-8.71)	3.35 (1.28-8.75)	5.25 (1.99-13.86)	
	p value^d^		0.017^*^	0.014^*^	0.001^**^	<0.001^**^
Hg	Control (%)	47 (24.74%)	48 (25.26%)	48 (25.26%)	47 (24.74%)	
	Case (%)	15 (10.95%)	39 (28.47%)	34 (24.82%)	49 (35.77%)	
	AOR (95% CI)	1	1.68 (0.72-3.89)	1.69 (0.72-3.96)	1.75 (0.76-4.03)	
	p value		0.228	0.226	0.185	0.004^**^
Pb	Control (%)	47 (24.74%)	48 (25.26%)	48 (25.26%)	47 (24.74%)	
	Case (%)	46 (33.58%)	19 (13.87)	17 (12.41%)	55 (40.15%)	
	AOR (95% CI)	1	0.37 (0.16-0.86)	0.16 (0.06-0.44)	0.90 (0.46-1.78)	
	p value		0.020^*^	< 0.001^**^	0.772	0.498
Cr	Control (%)	33 (24.81%)	34 (25.56%)	33 (24.81%)	33 (24.81%)	
	Case (%)	12 (15.79%)	14 (18.42%)	11 (14.47%)	39 (51.32%)	
	AOR (95% CI)	1	1.74 (0.51-5.96)	1.65 (0.45-6.10)	4.48 (1.40-14.31)	
	p value		0.377	0.450	0.011^*^	0.002^**^
Cd	Control (%)	22 (25.29%)	22 (25.29%)	22 (25.29%)	21 (24.14%)	
	Case (%)	5 (3.65%)	16 (11.68%)	64 (46.72%)	52 (37.96%)	
	AOR (95% CI)	1	3.07 (0.69-13.74)	16.87 (4.19-67.86)	11.95 (2.97-48.04)	
	p value		0.142	< 0.001^**^	< 0.001^**^	< 0.001^**^

^a^Missing values of Pb were imputed by 1/2 LOD while missing values of Cr and Cd were excluded from statistics. Quartiles based on the control group.

^b^Linear by linear association. ^*^ indicates p < 0.05, ^**^ indicates p < 0.01.

^c^AOR: Adjusted odds ratio. Adjustments made for maternal age, pre-pregnant BMI, gravidity, parity, HBV infection (HBsAg positive), newborn sex. CI: Confidence Interval.

^d^p value was obtained by binary logistic regression. ^*^ indicates p < 0.05, ^**^ indicates p < 0.01.

The dose-dependent relationship for Hg was noticeable before adjustment but not noticeable after adjustment. However, the trend for Hg kept significant. The significant AORs of 0.37 [95% CI 0.16, 0.86] and 0.16 [95% CI 0.06, 0.44] were observed for Pb in the 2^nd^ and 3^rd^ quartiles, respectively. Besides, the trend of linear association for Pb with GDM risk was not noticeable. These ambiguous trends may generally reflect inconclusive correlation between Pb, Hg and GDM risk.

## Discussion

### GDM incidence and some maternal risk factors

GDM incidences ranged from 1 to 14% worldwide [27]. In mainland of China, few studies reported the GDM incidence in different cities and explained possible risk factors. A large prospective study of 16286 pregnant women from 18 cities in China reported the GDM incidence was 4.3% [28], which is quite lower than that reported in current study (12%). This may be attributed towards distinct GDM risk factors at spatial scale. Therefore, the preliminary result from this nested case–control study suggested that the total 1359 participants might suffer from relatively higher GDM risk. In general, maternal age, pre-pregnant BMI, gravidity and parity, are the common GDM risk factors for Asian women [3-5,29], which are also observed in present study. The other definite risk factor is HBV infection, which had also been observed to increase the GDM risk in previous reports [25,26]. Moreover, our results also imply that the ‘diabetogenic environment of pregnancy’ may be further aggravated due to exposure to some heavy metals (i.e. As, Cd and Cr), in which these metals may act as obesogens to induce GDM risk [7-9,30].

### The gestational exposure to selected heavy metals

Heavy metals have been known to be able to transfer via placenta and penetrate into developing fetus by bioaccumulation [31-33]. Meconium is the fetal repository which traps the trans-placental xenobiotic residues during pregnancy and reflects exposure history. This matrix has been previously used to detect fetal exposure to a number of xenobiotic agents including drugs, alcohol metabolites, nicotine metabolites [14] and heavy metals (Table 4). However, only few studies addressed the maternal exposure by meconium [15,17]. In this study, the profiling of detected heavy metal residues in meconium suggested that the participants might commonly expose to these metals (Table 2). Compared to other reports, the extent of exposure may be area specific (Table 4), e.g., Pb, Hg and Cd exposure in inhabitants from heavily contaminated regions and/or industrialized metropolis [34-36] is noticeably higher than those from remote and/or rural areas [14,37,38]. Selected metals detected in our studied subjects may have similar sources, which could be mainly related to contaminated dietary items [39-41] and dust ingestion [42], as a consequence of rapid industrialization and increasing environmental pollution throughout China. Although no mother reported smoking during pregnancy, the second-hand cigarette smoking is very common in China. This may also be one of the routes for Cd exposure [43]. The levels of selected metals are higher in our subjects at many instances (Table 4), which clearly reflected severe environmental pollution in China.

**Table 4:** **Comparison of selected heavy metal levels in meconium (median, ng/g) with the published studies**

**Reference**	**Area**	**As**	**Hg**	**Pb**	**Cd**	**Cr**
The present work (n = 327)^a^	Xiamen, China	42.82	30.08	137.60	7.09	34.95
Türker et al., 2013 (n = 291) [37]	West Anatolia, Turkey	-	-	30.84	2.48	-
Vall et al., 2012 (n = 37) [44]	Tenerife Island, Spain	5.60	-	-	-	-
Gundacker et al., 2010 (n = 36) [38]	Vienna, Austria	-	4.00	15.50	-	-
Li et al., 2008 (n = 100) (mean) [34]	Guiyu, China	-	-	2.50 × 10^3^	-	-
Unuvar et al., 2007 (n = 143) (mean) [35]	Istanbul, Turkey	-	9.45 × 10^3^	-	-	-
Türker et al., 2006 (n = 117) [36]	Kocaeli, Turkey	-	-	4.65 × 10^4^	2.30 × 10^3^	-
Ostrea Jr. et al., 2002 (n = 426) (ppm) [14]	Manila, Philippines	0.00	3.17 × 10^−3^	35.77	13.37	-
Ramirez et al., 2000 (n = 36) (mean, ppm) [45]	Tagum, Philippines	-	4.86 × 10^−2^	-	-	-

^a^In the present work, missing values of Pb were imputed by 1/2 LOD. Cd values were available from 224 samples and Cr values were available from 209 samples.

### GDM risk from the selected heavy metals

In current study, we assess that heavy metal exposure may cause considerable risk of GDM prevalence in the investigated cohort, and the As exposure is quite noteworthy. Our results are in agreement with many other epidemiologic reports which linked diabetes mellitus to As exposure [46,47]. In some highly As-contaminated regions, the diabetes incidences could be two to five times higher when compared to the non-endemic regions [47]. Moreover, As also caused the maternal GDM risk [48] and previously a research has found a significant relationship between blood arsenic burden and impaired glucose tolerance in 532 US pregnant women [49]. Hence, our observations also support the hypothesis that As exposure may induce maternal GDM risk. Nevertheless, most studies were limited to inhabitants from the highly geogenic arsenic-contaminated areas; while some contradictory findings from less contaminated areas were justified by some other risk factors [12,50]. Similar effects have also been observed for Cr and Cd exposure in current study but their results are subjected to discrepant and low detection frequencies in the case–control samples, which may lead to some bias assessments. Cd exposure has been linked to diabetes risk in general population but the effects have not been confirmed yet [51,52]. Likewise, the role of trivalent Cr in controlling blood glucose is still controversial [53]. Thus, more detailed epidemiological studies are required to explore the effects of these metals. On the other hand, Pb and Hg have also been suggested to induce diabetes [54,55] and insulin resistance [56], respectively. However, the conclusions majorly came from special high-exposed populations, e.g. occupational workers [55] and Minamata disease patients [56].

### Strengths and limitations of this study

Our study provides the evidence that meconium is a novel non-invasive matrix for the surveillance of heavy metals during pregnancy, which can be mutually used to assess both prenatal children and gestational mothers’ exposure. In addition, meconium is formed in fetus 12 weeks earlier than maternal GDM diagnosis, which proves itself as an efficient tool to reflect the chronic exposure from the early pregnancy to the delivery. Our pilot work also showed the authentication of meconium as an effective monitoring tool in the large cohort study.

The quality of study design was ensured by following the universal GDM diagnostic criteria [19] with full consideration of confounders [47]. All potential cofounders, including gestational complications and clinical characteristics were carefully assessed between the GDM and non-GDM mothers (Additional file 1: Tables S2 and S3); and finally 6 risk factors were fully adjusted in GDM risk assessment. Our criteria to recruit controls were very stringent and we included only 190 apparently healthy mothers from 1193 non-GDM mothers. To identify potential selection bias in study group, clinical characteristics were compared between 327 selected mothers in nested case–control group and other 1032 non-selected mothers (Additional file 1: Table S8). Among all the potential risk factors, only parity and HBV infection were significantly different (*p < 0.05*) between selected and non-selected mothers but we believe that adjustments made for them have addressed this problem. Therefore, this nested case–control study would be fine-tuned to generally reflect the whole population’s condition.

Although GDM is not a rare disease, but the nested case–control sample size is still not large enough. Some missing clinical records led to 36 unavailable values of maternal age or pre-pregnant BMI in case group, which is an inherent limitation due to the nature of retrospective study. In addition, the low and discrepant detection of Cr and Cd may lead to bias and questionable results. Moreover, the species specific effects of Cr (i.e., highly toxic hexavalent but nutrient trivalent Cr) and As on the GDM development may reveal different effects. Therefore, the species specific measurements for these metals are expected in the further studies.

## Conclusions

Our pilot study showed the linkages of the selected heavy metal to maternal GDM risk, which implies that these heavy metals should be paid more attention. Although our finding supports the hypothesis that some metals (noticeably As) pose additional risk to GDM prevalence, more detailed toxicological information is required to support the observation. Meanwhile, our study provides the evidence that meconium is a useful matrix to assess both prenatal children and gestational mothers’ exposure to selected heavy metals during pregnancy.

## Additional file

Additional file 1: Text S1. Details of sample preparation and heavy metal analysis by ICP-MS. Text S2 QC/QA in the analytical procedure. **Table S1.** Calibration curves, linear range (LR), relative coefficients (r^2^) of calibration curves, limit of the detection (LOD) and the recoveries. **Table S2.** Complications of the mothers and newborns between GDM and non-GDM group in 1359 participants. **Table S3.** Clinical characteristics of the mothers and newborns in 1359 participants. **Table S4.** Clinical characteristics of the mothers and newborns in nested case–control group based on newborns’ sex. **Table S5.** Associations of Cr and Cd based on three cutoffs with adjusted odds ratio (AOR) of GDM in nested case–control group^a^. **Table S6.** Associations of heavy metals based on quartiles with crude odds ratio (OR) of GDM in nested case–control group^a^. **Table S7.** Associations of Cr and Cd based on three cutoffs with crude odds ratio (OR) of GDM in nested case–control group^a^. **Table S8.** Comparison of clinical characteristics between 327 selected subjects and other non-selected subjects.

## Abbreviations

GDM: Gestational diabetes mellitus
As: Arsenic
Hg: Mercury
Pb: Lead
Cd: Cadmium
Cr: Chromium
ICP-MS: Inductively coupled plasma mass spectrometry
LOD: Limit of detection
BMI: Body mass index
T2DM: Type 2 diabetes
IGT: Impaired glucose tolerance
OGTT: Oral glucose tolerance test
WHO: World Health Organization
HBV: Hepatitis B virus
CI: Confidence interval
OR: Odds ratio
AOR: Adjusted odds ratio
