# Supplementary material for: A nested case-control study indicating heavy metal residues in meconium associate with maternal gestational diabetes mellitus risk
Source: Environ Health. 2015 Feb 28;14:19. doi: 10.1186/s12940-015-0004-0 (PMC4357163; doi:10.1186/s12940-015-0004-0)
Supplement: Additional file 1: Text S1. — Details of sample preparation and heavy metal analysis by ICP-MS. Text S2 QC/QA in the analytical procedure. Table S1. Calibration curves, linear range (LR), relative coefficients (r2) of calibration curves, limit of the detection (LOD) and the recoveries. Table S2. Complications of the mothers and newborns between GDM and non-GDM group in 1359 participants. Table S3. Clinical characteristics of the mothers and newborns in 1359 participants. Table S4. Clinical characteristics of the mothers and newborns in nested case–control group based on newborns’ sex. Table S5. Associations of Cr and Cd based on three cutoffs with adjusted odds ratio (AOR) of GDM in nested case–control groupa. Table S6. Associations of heavy metals based on quartiles with crude odds ratio (OR) of GDM in nested case–control groupa. Table S7. Associations of Cr and Cd based on three cutoffs with crude odds ratio (OR) of GDM in nested case–control groupa. Table S8. Comparison of clinical characteristics between 327 selected subjects and other non-selected subjects. [file 12940_2015_4_MOESM1_ESM.docx]

**Do Heavy Metal Residues in Meconium Associate with Maternal Gestational Diabetes Mellitus Risk?**

**A Nested Case-Control Study**

**Text S1. Details of sample preparation and heavy metal analysis by ICP-MS**

Ultra-pure water was obtained from a Milli-Q system (Millipore Corporation, Billerica, MA, USA). All containers were soaked in 10% (v/v) HNO_3_ (Sinopharm Chemical Reagent Co., Ltd, Beijing, China) overnight and rinsed with ultra-pure water by three times. 100 mg dry meconium was carefully weighed for each sample, then digested with 1 mL GR grade 65% (v/v) HNO_3_ (CNW, Shanghai, China) overnight and then 1 mL GR grade 30% (v/v) H_2_O_2_ (Sinopharm Chemical Reagent Co., Ltd, Beijing, China) at the next day. The mixture was sealed and heated in a water bath at 100°C for at least 5 h until it turned totally transparent. All digested samples were filtered through a 0.22 μm nylon membrane and adjusted to a final volume of 5 mL with ultra-pure water. Some samples were spiked with Cr, As, Cd, Pb and Hg before digestion at the two final levels, i.e., 10 and 2 ng/mL for Cr, As, Cd, Pb and 2 and 0.5 ng/mL for Hg. The recoveries were measured by using these spiked samples.

The operating parameters of ICP-MS were set as RF power 1510W, carrier gas 1.1 L/min, makeup gas 0.10 L/min, helium gas 3.5 mL/min, nebulizer pump 0.1 rps. The analytical masses were ^53^Cr, ^75^As, ^111^Cd, ^202^Hg and ^208^Pb. The standard stock solution mixed with Cr, As, Cd and Pb (100µg/mL, GSB 04-1767-2004) was obtained from the National Center of Analysis and Testing for Nonferrous Metals and Electronic Materials (NCATN). Stock standard solution of Hg (100µg/mL, GBW(E) 080124) was obtained from the National Institute of Metrology. The working solutions were daily prepared by appropriate dilutions of standard stock solution using a mixture of 65% (v/v) HNO_3_, 30% (v/v) H_2_O_2_ and H_2_O (v/v/v=1:1:3).

**Text S2. QC/QA in the analytical procedure**

Relative percent difference for replicate analyses were < 5%. All samples were run in a randomized fashion (blinded to case-control status) to reduce the possible uncertainty from the artifacts related injection order and instrumental sensitivity change during in the entire sequence. All the containers used for the experiment were washed in one batch and all the samples were prepared and analyzed in the same analytic batch, too. The five selected metals concentrations of each sample were measured at the same analytic batch. A blank sample was prepared by undergoing the whole procedure without meconium. The procedure blank sample (without meconium) was also analyzed and none of the target metals was detected. A quality control (QC) sample was prepared by mixing aliquots of each sample and therefore broadly representative of the whole sample sets. The QC sample was injected in every 15 samples to assess the instrumental stability. The variations of all metal concentrations of QCs were < 15%. Meanwhile, 1 mg/L mixed internal standard was applied (Agilent, ICP-MS Internal Standard Mix, Bi, Ge, In, Li, Lu, Rh, Sc, Tb). The intensity of Sc, Ge, Rh, Tb and Lu kept almost steady through the experiment (RSD < 5%) suggested the sensibility and stability of signal.

**Table S1. Calibration curves, linear range (LR), relative coefficients (r^2^) of calibration curves, limit of the detection (LOD) and the recoveries.**

| Element | Calibration curve ^a^ | LR | r^2^ | LOD | Recovery (%) ^b^ | |
| --- | --- | --- | --- | --- | --- | --- |
|  |  |  |  |  | High Level | Low Level |
| Cr | Y=8.41×10^-3^ X+7.80×10^-3^ | 0.25-25.00 | 0.9996 | 0.12 | 93.16 ± 9.06 | 79.07 ± 1.13 |
| As | Y=2.84×10^-3^ X+3.47×10^-4^ | 0.25-25.00 | 0.9999 | 0.06 | 114.54 ± 2.57 | 127.80 ± 16.45 |
| Cd | Y=1.07×10^-3^ X+2.00×10^-4^ | 0.25-25.00 | 0.9999 | 0.03 | 109.91 ± 2.70 | 115.04 ± 3.12 |
| Hg | Y=1.12×10^-3^ X+8.98×10^-6^ | 0.10-5.00 | 1.0000 | 0.01 | 98.82 ± 4.30 | 107.59 ± 1.80 |
| Pb | Y=1.80×10^-2^ X+1.75×10^-2^ | 0.25-25.00 | 0.9993 | 0.13 | 100.16 ± 9.51 | 121.06 ± 10.92 |

^a^ X represents concentration (ng/mL); Y represents ratio (intensity adjusted by internal standard).

^b^ Values are expressed as mean ± SD (n=4). Spiked high level was 10 ng/mL for Cr, As, Cd, Pb and 2ng/mL for Hg; Spiked low level was 2 ng/mL for Cr, As, Cd, Pb and 0.5 ng/mL for Hg

**Table S2. Complications of the mothers and newborns between GDM and non-GDM group in 1359 participants.**

|  | GDM (n=166) | non-GDM (n=1193) | p^a^ |
| --- | --- | --- | --- |
| **Maternal gestational complications** | | | |
| HBsAg positive | 28 (17.28%) | 134 (11.74%) | 0.046^*^ |
| Binocular ametropia | 3 (1.81%) | 12 (1.01%) | 0.414 |
| B group hemolytic streptococcus infection of genital tract | 4 (2.41%) | 14 (1.17%) | 0.263 |
| Trichomonas vaginitis | 0 (0.00%) | 1 (0.08%) | 1.000 |
| Upper respiratory infections | 0 (0.00%) | 2 (0.17%) | 1.000 |
| Chorioamnionitis | 0 (0.00%) | 2 (0.17%) | 1.000 |
| Ankylosing spondylitis | 1 (0.60%) | 0 (0.00%) | 0.122 |
| Epilepsy | 0 (0.00%) | 1 (0.08%) | 1.000 |
| Hypophysoma | 1 (0.60%) | 0 (0.00%) | 0.122 |
| Diabetes mellitus | 0 (0.00%) | 1 (0.08%) | 1.000 |
| Pre-eclampsia | 2 (1.20%) | 6 (0.50%) | 0.255 |
| Gestational hypertension | 1 (0.60%) | 10 (0.84%) | 1.000 |
| Impaired liver function | 1 (0.60%) | 2 (0.17%) | 0.324 |
| Heart disorders^b^ | 1 (0.60%) | 8 (0.67%) | 1.000 |
| Cholestasis | 3 (1.81%) | 9 (0.75%) | 0.172 |
| Thyroid function disorders^c^ | 2 (1.20%) | 8 (0.67%) | 0.350 |
| Renal disorders^d^ | 0 (0.00%) | 2 (0.17%) | 1.000 |
| Oophoritic cyst | 2 (1.20%) | 2 (0.17%) | 0.075 |
| General anemia | 4 (2.41%) | 17 (1.42%) | 0.312 |
| Thalassemia | 4 (2.41%) | 28 (2.35%) | 1.000 |
| Gestational thrombocytopenia | 1 (0.60%) | 5 (0.42%) | 0.543 |
| Cicatricial uterus | 5 (3.01%) | 45 (3.77%) | 0.626 |
| Hysteromyoma | 1 (0.60%) | 20 (1.68%) | 0.501 |
| Uterus duplex | 1 (0.60%) | 1 (0.08%) | 0.229 |
| Cervical laceration | 1 (0.60%) | 11 (0.92%) | 1.000 |
| Uterus malformation^e^ | 2 (1.20%) | 2 (0.17%) | 0.075 |
| Twin pregnancy | 3 (1.81%) | 12 (1.01%) | 0.414 |
| Pregnancy with intra-uterine device (IUD) in situ | 1 (0.60%) | 1 (0.08%) | 0.229 |
| Prolonged pregnancy | 0 (0.00%) | 2 (0.17%) | 1.000 |
| Prolonged second stage of labor | 0 (0.00%) | 3 (0.25%) | 1.000 |
| Fetal distress in uterus | 8 (4.82%) | 55 (4.61%) | 0.904 |
| Fetal growth restriction in uterus | 0 (0.00%) | 1 (0.08%) | 1.000 |
| Oligohydramnios | 5 (3.01%) | 62 (5.20%) | 0.223 |
| Hydramnion | 4 (2.41%) | 12 (1.01%) | 0.121 |
| Premature rupture of fetal membranes | 28 (16.87%) | 226 (18.94%) | 0.520 |
| Umbilical cord disorders^f^ | 74 (44.58%) | 416 (34.87%) | 0.015^*^ |
| Placental disorders^g^ | 9 (5.42%) | 49 (4.11%) | 0.432 |
| Relative cephalopelvic disproportion | 8 (4.82%) | 43 (3.60%) | 0.440 |
| Malposition | 6 (3.61%) | 35 (2.93%) | 0.631 |
| General contracted pelvis | 0 (0.00%) | 1 (0.08%) | 1.000 |
| Premature birth | 5 (3.01%) | 18 (1.51%) | 0.188 |
| Precipitate labor | 18 (10.84%) | 32 (2.68%) | < 0.001^**^ |
| Shoulder dystocia | 0 (0.00%) | 1 (0.08%) | 1.000 |
| Vaginal wall damage | 2 (1.20%) | 8 (0.67%) | 0.350 |
| Postpartum hemorrhage | 1 (0.60%) | 14 (1.17%) | 1.000 |
| Maternal-fetal blood group incompatibility | 3 (1.81%) | 19 (1.59%) | 0.744 |
| Chromosome abnormalities | 0 (0.00%) | 1 (0.08%) | 1.000 |
| Congenital blindness of the right eye | 0 (0.00%) | 1 (0.08%) | 1.000 |
| **Neonatal disorders** | | | |
| Macrosomia | 4 (2.41%) | 39 (3.27%) | 0.553 |
| Small for date infant | 2 (1.20%) | 20 (1.68%) | 1.000 |
| Precious infant | 1 (0.60%) | 1 (0.08%) | 0.229 |
| Birth defects^h^ | 0 (0.00%) | 4 (0.34%) | 1.000 |
| Fetal left hydronephrosis | 0 (0.00%) | 2 (0.17%) | 1.000 |
| Areflexia | 0 (0.00%) | 1 (0.08%) | 1.000 |
| Single umbilical artery | 0 (0.00%) | 1 (0.08%) | 1.000 |
| Fetal chromosomal abnormalities | 0 (0.00%) | 1 (0.08%) | 1.000 |

^a^ Fisher’s exact test was applied for factors with total observed frequency ≤ 40. Otherwise, Pearson Chi-square test was applied.

^*^ indicates p < 0.05, ^**^ indicates p < 0.01.

^b^ Heart disorders includes arrhythmia, nodal tachycardia, repair of ventricular septal defect, complete right bundle branch block.

^c^ Thyroid function disorders includes hyperthyroidism and hypothyroidism.

^d^ Renal disorders includes glomerulonephritis, renal calculus and uronephrosis.

^e^ Uterus malformation includes mediastinal uterus, uterus unicornis, and uterus subseptus.

^f^ Umbilical cord disorders includes torsion of umbilical cord, false/true knot of umbilical cord, long/short cord, cord around neck, cord entanglement and recessive prolapse of umbilical cord.

^g^ Placental disorders includes placental abruption, velamentous placenta, battledore placenta, complete placenta previa, low-lying placenta, accessory placenta and adherent placenta.

^h^ Birth defects includes hexactylia, persistent left superior vena cava and hypospadias.

**Table S3 Clinical characteristics of the mothers and newborns in 1359 participants.**

|  | GDM (n=166) | Non-GDM (n=1193) | p^a^ |
| --- | --- | --- | --- |
| Newborn birth weight (kg)^b^ | 3.27 ± 0.38 | 3.24 ± 0.39 | 0.245 |
| Maternal age (year)^c^ | 27.94 ± 3.99 | 26.76 ± 3.24 | 0.001^**^ |
| Maternal pre-pregnant BMI (kg/m^2^)^d^ | 22.06 ± 3.14 | 20.91 ± 2.59 | < 0.001^**^ |
| Gestational age (week)^e^ | 39.10 ± 0.85 | 39.45 ± 1.11 | < 0.001^**^ |
| Newborn sex | | | |
| Male | 86 (51.81%) | 643 (53.90%) | 0.613 |
| Female | 80 (48.19%) | 550 (46.10%) |  |
| Maternal gravidity^f^ | | | |
| 1 | 97 (59.15%) | 743 (62.33%) | 0.638 |
| 2 | 40 (24.39%) | 283 (23.74%) |  |
| 3 | 19 (11.59%) | 103 (8.64%) |  |
| ≥4 | 8 (4.88%) | 63 (5.29%) |  |
| Maternal parity^g^ | | | |
| 1 | 137 (83.54%) | 993 (83.38%) | 0.922 |
| 2 | 26 (15.85%) | 187 (15.70%) |  |
| ≥3 | 1 (0.61%) | 11 (0.92%) |  |
| Mode of delivery | | | |
| Eutocia | 113 (68.07%) | 919 (77.03%) | 0.038^*^ |
| C-section | 46 (27.71%) | 242 (20.28%) |  |
| Aid delivery | 7 (4.22%) | 32 (2.68%) |  |

^a^ p value of continuous variable was obtained by non-parametric Mann-Whitney U test, while p value of categorical variable was obtained by Pearson Chi-square test.

^*^ indicates p < 0.05, ^**^ indicates p < 0.01.

^b^ Values from 164 subjects in GDM group and 1191 subjects in non-GDM group were available. Valuesare expressed as mean ± SD.

^c^ Values from 124 subjects in GDM group and 914 subjects in non-GDM group were available. Values are expressed as mean ± SD.

^d^ Values from 126 subjects in GDM group and 925 subjects in non-GDM group were available. Values are expressed as mean ± SD.

^e^ Gestational age: A measure of the age of a pregnancy where the origin is the woman's last normal menstrual period (LMP). Values are expressed as mean ± SD.

^f^ Gravidity: Number of times a woman has been pregnant, regardless of whether the pregnancies were interrupted or resulted in a live birth.

^g^ Parity: The number of pregnancies carried to viable gestational age.

**Table S4. Clinical characteristics of the mothers and newborns in nested case-control group based on newborns’ sex.**

|  | Male | | | Female | | |
| --- | --- | --- | --- | --- | --- | --- |
|  | Case (n=72) | Control (n=93) | p^b^ | Case (n=65) | Control (n=97) | p^c^ |
| Newborn birth weight (kg)^a^ | 3.36 ± 0.39 | 3.28 ± 0.29 | 0.162 | 3.23 ± 0.36 | 3.18 ± 0.31 | 0.326 |
| Maternal age (year)^d^ | 28.39 ± 4.37 | 26.23 ± 2.69 | 0.001^**^ | 27.30 ± 3.23 | 26.44 ± 2.60 | 0.084 |
| Maternal pre-pregnant BMI (kg/m^2^)^e^ | 21.95 ± 3.14 | 20.87 ± 2.38 | 0.049^*^ | 21.83 ± 2.72 | 20.31 ± 2.13 | 0.001^**^ |
| Gestational age (week)^f^ | 39.15 ± 0.81 | 39.48 ± 0.98 | 0.030^*^ | 39.22 ± 0.79 | 39.62 ± 0.86 | 0.007^*^ |
| Maternal gravidity | | | | | | |
| 1 | 39 (54.17%) | 68 (73.12%) | 0.014^*^ | 43 (66.15%) | 66 (68.04%) | 0.502 |
| 2 | 19 (26.39%) | 19 (20.43%) |  | 10 (15.38%) | 19 (19.59%) |  |
| ≥3 | 14 (19.44%) | 6 (6.45%) |  | 12 (18.46%) | 12 (12.37%) |  |
| Maternal parity | | | | | | |
| 1 | 58 (80.56%) | 85 (91.40%) | 0.042^*^ | 56 (86.15%) | 89 (91.75%) | 0.320 |
| 2 | 14 (19.44%) | 8 (8.60%) |  | 8 (12.31%) | 8 (8.25%) |  |
| ≥3 | 0 (0.00%) | 0 (0.00%) |  | 1 (1.54%) | 0 (0.00%) |  |
| Mode of delivery | | | | | | |
| Eutocia | 45 (62.50%) | 85 (91.40%) | < 0.001^**^ | 47 (72.31%) | 91 (93.81%) | 0.001^**^ |
| C-section | 23 (31.94%) | 7 (7.53%) |  | 16 (24.62%) | 6 (6.19%) |  |
| Aid delivery | 4 (5.56%) | 1 (1.08%) |  | 2 (3.08%) | 0 (0.00%) |  |
| Maternal HBsAg | | | | | | |
| Positive | 15 (21.13%) | 0 (0.00%) | < 0.001^**^ | 9 (13.85%) | 0 (0.00%) | < 0.001^**^ |
| Negative | 56 (78.87%) | 93 (100.00%) |  | 56 (86.15%) | 97 (100.00%) |  |

^a-f^ Values are expressed as Mean ± SD.

^b^ p Values of maternal age, pre-pregnant BMI, gestational age were obtained by non-parametric Mann-Whitney U test while of newborn birth weight was obtained by independent samples t-test; p values of gravidity and parity, mode of delivery were obtained by Chi-square test; p values of HBsAg, were obtained by Fisher’s exact test.

^c^ p Values of maternal pre-pregnant BMI, gestational age were obtained by non-parametric Mann-Whitney U test; p values of newborn birth weight and maternal age were obtained by independent samples t-test; p Values of gravidity and parity, mode of delivery were obtained by Chi-square test; p values of HBsAg was obtained by Fisher’s exact test.

^b c *^ indicates p < 0.05, ^**^ indicates p < 0.01

^d^ In male newborn group, values of maternal age were available from 51 subjects in case group; In female newborn group, values of maternal age were available from 50 subjects in case group.

^e^ In male newborn group, values of maternal pre-pregnant BMI age were available from 52 subjects in case group; In female newborn group, values of maternal age were available from 51 subjects in case group.

**Table S5. Associations of Cr and Cd based on three cutoffs with adjusted odds ratio (AOR) of GDM in nested case-control group^a^.**

| **Metal** | **Control and Case** | **1st Cutoff (Reference)** | **2nd Cutoff** | **3rd Cutoff** | **p trend^d^** |
| --- | --- | --- | --- | --- | --- |
| **Cr** | Control (%) | 67 (50.38%) | 33 (24.81%) | 33 (24.81%) |  |
|  | Case (%) | 26 (34.21%) | 11 (14.47%) | 39 (51.32%) |  |
|  | AOR (95% CI)^b^ | 1 | 1.20 (0.41-3.51) | 3.27 (1.35-7.93) |  |
|  | p value^c^ |  | 0.733 | 0.009^**^ | 0.001^**^ |
| **Cd** | Control (%) | 44 (50.57%) | 22 (25.29%) | 21 (24.14%) |  |
|  | Case (%) | 21 (15.33%) | 64 (46.72%) | 52 (37.96%) |  |
|  | AOR (95% CI)^b^ | 1 | 8.26 (3.39-20.12) | 5.82 (2.39-14.16) |  |
|  | p value^c^ |  | < 0.001^**^ | < 0.001^**^ | < 0.001^**^ |

^a^ Missing values of Cr and Cd were excluded from statistics. Cutoffs based on cutoff-points at 50% and 75% of the controls.

^b^ AOR: Adjusted odds ratio. Adjustments made for maternal age, pre-pregnant BMI, gravidity, parity, HBV infection (HBsAg positive), newborn sex.

^c^ p value was obtained by binary logistic regression. ^*^ indicates p < 0.05, ^**^ indicates p < 0.01.

^d^ Linear by linear association. ^*^ indicates p < 0.05, ^**^ indicates p < 0.01.

**Table S6. Associations of heavy metals based on quartiles with crude odds ratio (OR) of GDM in nested case-control group^a^.**

| **Metal** | **Control and Case** | **1st Quartile (Reference)** | **2nd Quartile** | **3rd Quartile** | **4th Quartile** | **p trend^c^** |
| --- | --- | --- | --- | --- | --- | --- |
| **As** | Control (%) | 47 (24.74%) | 48 (25.26%) | 48 (25.26%) | 47 (24.74%) |  |
|  | Case (%) | 11 (8.03%) | 32 (23.36%) | 44 (32.12%) | 50 (36.50%) |  |
|  | OR (95% CI) | 1 | 2.85 (1.29-6.30) | 3.92 (1.81-8.49) | 4.55 (2.11-9.80) |  |
|  | p value^b^ |  | 0.010^*^ | 0.001^**^ | < 0.001^**^ | < 0.001^**^ |
| **Hg** | Control (%) | 47 (24.74%) | 48 (25.26%) | 48 (25.26%) | 47 (24.74%) |  |
|  | Case (%) | 15 (10.95%) | 39 (28.47%) | 34 (24.82%) | 49 (35.77%) |  |
|  | OR (95% CI) | 1 | 2.55 (1.24-5.22) | 3.22 (1.07-4.60) | 3.27 (1.61-6.62) |  |
|  | p value |  | 0.011^*^ | 0.032^*^ | 0.001^**^ | 0.004^**^ |
| **Pb** | Control (%) | 47 (24.74%) | 48 (25.26%) | 48 (25.26%) | 47 (24.74%) |  |
|  | Case (%) | 46 (33.58%) | 19 (13.87) | 17 (12.41%) | 55 (40.15%) |  |
|  | OR (95% CI) | 1 | 0.40 (0.21-0.79) | 0.36 (0.18-0.72) | 1.20 (0.68-2.10) |  |
|  | p value |  | 0.008^**^ | 0.004^**^ | 0.534 | 0.498 |
| **Cr** | Control (%) | 33 (24.81%) | 34 (25.56%) | 33 (24.81%) | 33 (24.81%) |  |
|  | Case (%) | 12 (15.79%) | 14 (18.42%) | 11 (14.47%) | 39 (51.32%) |  |
|  | AOR (95% CI) | 1 | 1.13 (0.46-2.81) | 0.92 (0.35-2.37) | 3.25 (1.45-7.28) |  |
|  | p value |  | 0.788 | 0.858 | 0.004^**^ | 0.002^**^ |
| **Cd** | Control (%) | 22 (25.29%) | 22 (25.29%) | 22 (25.29%) | 21 (24.14%) |  |
|  | Case (%) | 5 (3.65%) | 16 (11.68%) | 64 (46.72%) | 52 (37.96%) |  |
|  | OR (95% CI) | 1 | 3.20 (1.00-10.26) | 12.80 (4.32-37.89) | 10.90 (3.64-32.58) |  |
|  | p value |  | 0.05 | < 0.001^**^ | < 0.001^**^ | < 0.001^**^ |

^a^ Missing values of Pb were imputed by 1/2 LOD while missing values of Cr and Cd were excluded from statistics. Quartiles based on the control group.

^b^ p value was obtained by binary logistic regression. ^*^ indicates p < 0.05, ^**^ indicates p < 0.01.

^c^ Linear by linear association. ^*^ indicates p < 0.05, ^**^ indicates p < 0.01.

**Table S7. Associations of Cr and Cd based on three cutoffs with crude odds ratio (OR) of GDM in nested case-control group^a^.**

| **Metal** | **Control and Case** | **1st Cutoff (Reference)** | **2nd Cutoff** | **3rd Cutoff** | **p trend^c^** |
| --- | --- | --- | --- | --- | --- |
| **Cr** | Control (%) | 67 (50.38%) | 33 (24.81%) | 33 (24.81%) |  |
|  | Case (%) | 26 (34.21%) | 11 (14.47%) | 39 (51.32%) |  |
|  | OR (95% CI) | 1 | 0.86 (0.38-1.95) | 3.05 (1.59-5.82) |  |
|  | p value^b^ |  | 0.716 | 0.001^**^ | 0.001^**^ |
| **Cd** | Control (%) | 44 (50.57%) | 22 (25.29%) | 21 (24.14%) |  |
|  | Case (%) | 21 (15.33%) | 64 (46.72%) | 52 (37.96%) |  |
|  | OR (95% CI) | 1 | 6.10 (3.00-12.40) | 5.19 (2.51-10.72) |  |
|  | p value^b^ |  | < 0.001^**^ | < 0.001^**^ | < 0.001^**^ |

^a^ Missing values of Cr and Cd were excluded from statistics. Cutoffs based on cutoff-points at 50% and 75% of the controls.

^b^ p value was obtained by binary logistic regression. ^*^ indicates p < 0.05, ^**^ indicates p < 0.01.

^c^ Linear by linear association. ^*^ indicates p < 0.05, ^**^ indicates p < 0.01.

**Table S8 Comparison of clinical characteristics between 327 selected subjects and other non-selected subjects.**

|  | **Selected (n=327)** | **Non-selected (n=1032)** | **p^a^** |
| --- | --- | --- | --- |
| **Newborn birth weight (kg)^b^** | 3.25 ± 0.34 | 3.24 ± 0.40 | 0.431 |
| **Maternal age (year)^c^** | 26.90 ± 3.25 | 26.90 ± 3.40 | 0.912 |
| **Maternal pre-pregnant BMI (kg/m2)^d^** | 21.07 ± 2.63 | 21.04 ± 2.71 | 0.721 |
| **Gestational age (week)^e^** | 39.40 ± 0.88 | 39.41 ± 1.15 | 0.442 |
| **Newborn sex** | | | |
| Male | 165 (50.15%) | 564 (54.76%) | 0.145 |
| Female | 164 (49.85%) | 466 (45.24%) |  |
| **Maternal gravidity** | | | |
| 1 | 217 (66.36%) | 623 (60.54%) | 0.065 |
| 2 | 68 (20.80%) | 255 (24.78%) |  |
| 3 | 32 (9.79%) | 90 (8.75%) |  |
| ≥4 | 10 (3.06%) | 61 (5.93%) |  |
| **Maternal parity** | | | |
| 1 | 288 (88.07%) | 841 (81.81%) | 0.008^**^ |
| 2 | 38 (11.62%) | 175 (17.02%) |  |
| ≥3 | 1 (0.31%) | 12 (1.17%) |  |
| **Mode of delivery** | | | |
| Eutocia | 270 (82.07%) | 762 (73.98%) | 0.012^*^ |
| C-section | 52 (15.81%) | 236 (22.19%) |  |
| Aid delivery | 7 (2.13%) | 32 (3.11%) |  |
| **Maternal HBsAg** | | | |
| Positive | 24 (7.32%) | 138 (14.15%) | 0.001^**^ |
| Negative | 304 (92.68%) | 837 (85.85%) |  |

^a^ p Values of newborn birth weight, maternal age, pre-pregnant BMI, gestational age were obtained by non-parametric Mann-Whitney U test while p values of newborn sex, maternal gravidity and parity, mode of delivery, HBsAg were obtained by Chi-square test;

^*^ indicates p < 0.05, ^**^ indicates p < 0.01

^b-e^ Values are expressed as Mean ± SD.

^b^ Values of newborn birth weight were available from 1028 subjects in non-selected group.

^c^ Values of maternal age were available from 291 subjects in selected group and 746 subjects in non-selected group.

^d^ Values of maternal pre-pregnant BMI were available from 293 subjects in selected group and 757 subjects in non-selected group.
